# Need of cost-effective vaccines in developing countries: What plant biotechnology can offer?

**DOI:** 10.1186/s40064-016-1713-8

**Published:** 2016-01-22

**Authors:** Mohammad Tahir Waheed, Muhammad Sameeullah, Faheem Ahmed Khan, Tahira Syed, Manzoor Ilahi, Johanna Gottschamel, Andreas Günter Lössl

**Affiliations:** Department of Biochemistry, Faculty of Biological Sciences, Quaid-i-Azam University, Islamabad, 45320 Pakistan; Department of Horticulture, Faculty of Agriculture and Natural Sciences, Abant Izzet Baysal University, Golkoy Campus, 14280 Bolu, Turkey; Molecular Biotechnology Laboratory for Triticeae Crops, Huazhong Agricultural University, Wuhan, China; NIBIO - Norwegian Institute of Bioeconomy Research, Ås, Norway; Department of Applied Plant Sciences and Plant Biotechnology, University of Natural Resources and Applied Life Sciences, Konrad Lorenz Straße 24, 3430 Tulln an der Donau, Austria; AIT Austrian Institute of Technology GmbH, Donau-City-Straße 1, 1220 Vienna, Austria

**Keywords:** Biopharmaceuticals, Plant-based vaccines, Adjuvants, Infectious diseases, Drug resistant microorganisms, Next generation vaccines

## Abstract

To treat current infectious diseases, different therapies are used that include drugs or vaccines or both. Currently, the world is facing an increasing problem of drug resistance from many pathogenic microorganisms. In majority of cases, when vaccines are used, formulations consist of live attenuated microorganisms. This poses an additional risk of infection in immunocompromised patients and people suffering from malnutrition in developing countries. Therefore, there is need to improve drug therapy as well as to develop next generation vaccines, in particular against infectious diseases with highest mortality rates. For patients in developing countries, costs related to treatments are one of the major hurdles to reduce the disease burden. In many cases, use of prophylactic vaccines can help to control the incidence of infectious diseases. In the present review, we describe some infectious diseases with high impact on health of people in low and middle income countries. We discuss the prospects of plants as alternative platform for the development of next-generation subunit vaccines that can be a cost-effective source for mass immunization of people in developing countries.

## Background

The current global population has reached 7.3 billion and is expected to exceed 9 billion by 2050 (Population Reference Bureau 2015). Most of this increase will occur in developing countries, particularly in low income countries. Among these countries, the largest increase will be in Africa, where the population is expected to approximately double from 1.1 to 2.3 billion (Population Reference Bureau 2015). Due to this increase in population, people in the developing countries are expected to get more seriously effected compared to industrially developed countries in terms of resources, poverty and diseases.

In developing countries, main factors responsible for increased burden of diseases and mortality include poverty, poor sanitation, lack of proper health care services and non-affordability of costly drugs. Approximately, 1.3 billion people, which is one-third of the population, have income less than 1$ in developing world. Consequently, these people have less money to spend on healthcare. Additionally, war, terrorism as well as economic and social instabilities lead to mass displacement of people in different parts of the world, which makes not only these people vulnerable to infectious diseases, but also can cause the spread of diseases to other areas.

Although many diseases have been controlled in most parts of the world, re-emergence of some diseases such as polio still poses a threat. There are many obstacles in controlling the communicable diseases in developing countries. Among these are high prices of drugs and pharmaceuticals, resistance of microorganisms to drugs and lack of development of new drugs. There are number of diseases such as rabies, influenza, cholera and some relatively new challenges like Ebola that can locally cause high mortality. In the present review, we will briefly discuss several infectious diseases that are responsible for approximately 90 % of deaths worldwide, mostly in developing countries (WHO 1999). Current preventive strategies against these diseases will be given and the potential advantages of plant-based systems for the expression of vaccine antigens will be discussed. Prominent plant-based vaccine candidates expressed against these infectious diseases will also be described.

## Malaria

Malaria is caused by plasmodium species. It is reported that approximately 3.2 billion people are at risk of being infected with malaria worldwide. Among these, 1.2 billion are at high risk. The latest estimates show that there were approximately 198 million cases of malaria and 584,000 deaths in 2013 (WHO 2015a). Region with highest disease burden is Africa where 90 % of all malaria-related deaths occur. Mortality rate in this region is very high among children where one child dies of malaria every minute. Although death rate due to malaria has decreased in past decade, many developing countries are still affected. Currently, chemotherapy is used to combat this deadly disease. However, plasmodium has developed resistance to the currently available and affordable drugs. For instance, *Plasmodium falciparum*, first documented in Southeast Asia and spreading to Africa, developed resistance to chloroquine, (Wellems and Plowe 2001). Resistance of plasmodium to other chemotherapeutic agents is also reported (Bell and Winstanley 2004; Horn and Duraisingh 2014). Use of antimalarial drugs is effective but the development of drug resistance by plasmodium requires multidrug treatment (Bell and Winstanley 2004) which makes the development of affordable and effective treatment challenging. To develop an effective combat strategy and avoid the chemotherapy-related side effects, development of vaccine against malaria would be more suitable.

There are three distinct stages that can be used to develop vaccines against malaria; liver, blood and mosquito. A number of approaches have been adapted to develop subunit vaccines using antigens either from pre-erythrocytic or sexual stage parasites; the later more commonly called as transmission blocking vaccines (TBVs). Currently, no vaccine is available in market against malaria. However, there are number of vaccine candidates that are assessed in clinical trials (Schwartz et al. 2012; Draper et al. 2015; http://www.who.int/vaccine_research/links/Rainbow/en/index.html). These vaccine candidates are expressed in yeast, bacteria, insect and/or mammalian cells, which are costly production platforms. If a vaccine candidate successfully passes through the trials, its large scale inexpensive production will still be questionable. In contrast, plant-based systems would be helpful to develop cost-effective and scalable production of vaccines against malaria. Many antimalarial vaccine antigens have been expressed in plants, recently reviewed by Gregory and Mayfield (2014) and Chan and Daniell (2015). There are number of interesting recent reports of expression of TBV candidates in plants. One of these reports is from Biess et al. (2015) who expressed fusion protein F0 comprising Pfs25 and the C0-domain of Pfs230 in *Nicotiana benthamiana*. Upon immunization, antibodies were raised in mice against plant-derived F0 that completely blocked the formation of oocysts in malaria transmission-blocking assay (TBA) making F0 an interesting TBV candidate or a component of a multi-stage malaria vaccine cocktail. Jones et al. (2015) produced Pfs25 fused to a modified lichenase (LicKM) carrier in *N. benthamiana*. The antigen induced transmission blocking antibodies that persisted for up to 6 months after immunization in mice and rabbits. A plant-produced antigen Pfs25, combined with alhydrogel as an adjuvant is under investigation in phase I clinical trials (https://clinicaltrials.gov/ct2/show/NCT02013687?term=Pfs25&rank=4).

## Dengue

Although first recognized in 1950s in Philippines and Thailand, dengue has spread throughout the world and become a major international health concern. Over 2.5 billion people are at risk of dengue and WHO estimates that there may be 50–100 million cases every year worldwide. In a recent report, it is estimated that this number could be three times more than WHO estimates, up to 390 million new cases every year (Bhatt et al. 2013). Dengue is caused by four distinct but closely related viral serotypes, DENV1-DENV4. Recovery after the infection from one serotype provides immunity against only that particular serotype and cross protectivity against other serotypes is only partial and temporary. Currently there is no vaccine for the treatment of dengue. There are few vaccines which are under development against dengue including attenuated, recombinant, subunit, chimeric and DNA vaccines (Saejung et al. 2007). However, production costs related to these vaccine candidates still raise the question of their availability in low-income countries (Mahoney et al. 2012). Three viral proteins prM, E and NS1 are considered prime candidate antigens for subunit vaccine development against dengue. Saejung et al. (2007) expressed domain III of dengue 2-envelope protein (D2EIII, 298–400 amino acids) was successfully expressed in *N. benthamiana* cells using a TMV-based transient expression system. Mice intramuscularly immunized with the plant-derived antigen elicited anti-dengue humoral responses. EIII domain has also been expressed in plants in fusion with cholera toxin subunit (CTB), although the expression was very low (Kim et al. 2010). Some recent reports show the expression of dengue virus domain III in maize seeds and *Cucurbita pepo* (Kim and Yang 2014; Libsittikul et al. 2015), NS1 in tobacco (Amaro et al. 2015) and envelope glycoprotein in rice calli (Kim et al. 2014). However, immunogenicity of these candidates still needs to be tested in animal models.

## Human immunodeficiency virus (HIV) infection and acquired immunodeficiency syndrome (AIDS)

HIV continues to be one of the leading infectious agents accounting for approximately more than 34 million deaths so far. It is estimated that in 2014, approximately 1.2 million people died due to HIV-related causes throughout the world (WHO 2015b). Globally, approximately 36.9 million people were living with HIV at the end of 2014. Although new HIV infections have fallen by 35 % since 2000, approximately 2 million people were infected with HIV in 2014 (UNAIDS 2015). The impact of HIV has been devastating in many developing countries; more than 20 % of adults are infected in some parts of Africa (Mabey 2014). The most affected area is the Sub-Saharan Africa that accounts for almost 70 % of the global total of new HIV infections and the statistics show that ~25.8 million people were living with HIV in 2014 in this region (WHO 2015b). There is no cure to HIV and the infection can lead to AIDS, a condition in which there is a loss of body’s cellular immunity that greatly reduces the resistance to other infections and malignancies. However, current effective treatment with antiretroviral (ARV) drugs can control the virus. Although effective care and preventive strategies together with antiretroviral therapies have reduced the rates of HIV/AIDS and mortality levels have declined overtime, an effective vaccine is needed to complement existing strategies.

It is estimated that approximately 600 million US dollars per year are invested in HIV vaccine research, the majority from the US National Institutes of Health. However, not enough is being spent to develop candidate vaccines based on HIV subtypes circulating in developing countries, or to strengthen vaccine evaluation sites in these countries where most infections and deaths occur. Many vaccine antigens targeting HIV has been expressed in plants. These include env, Pr55Gag, p17, p25, gp41 and tat antigens (Rosales-Mendoza et al. 2014; Chan and Daniell 2015). These plant-derived vaccine candidates against HIV have shown very promising results in animal models inducing humoral and cellular responses. A recent report (Rubio-Infante et al. 2015) shows an effective approach for developing a multi-HIV vaccine consisting of a multi-epitopic protein comprising of the C4, V1, V2, V3 domains and the ELDKWA epitope derived from the gp120 and gp41 envelope proteins of HIV, respectively, expressed in tobacco chloroplasts. Multi-epitopic protein was orally administered to mice and specific IFN-γ production was observed in both CD4+ and CD8+ T cells showing T helper specific responses.

## Tuberculosis

Tuberculosis (TB), caused by *Mycobacterium tuberculosis*, remains one of the world’s deadliest communicable diseases and the second most frequent cause of death due to one single pathogen. It is estimated that almost one-third population of the world is infected with *Mycobacterium*. In 2013, an estimated 9 million people developed TB and 1.5 million died from the disease (WHO 2014). Of these 9 million people, more than half (56 %) belonged to South-East Asia and Western Pacific regions. Further one quarter cases occurred in the African Region. Overall, 95 % of new cases and deaths occur in developing countries. Current treatment strategies include drugs including antibiotics. However, *Mycobacterium* has developed resistance to multiple drugs and treatment of TB with these drugs is becoming difficult (Koul et al. 2011). In addition, development of much needed new drugs against TB is also not getting much share from pharmaceutical industry. For instance, in 2013, spending on tuberculosis by pharmaceutical companies was just $99 million (Zumla et al. 2015). The lone existing BCG vaccine, consisting of attenuated bacteria, provides only limited protection during childhood and has less or no effect on the rate of pulmonary diseases in adults (Kaufmann et al. 2010; Gengenbacher and Kaufmann 2012). Few vaccines against TB are under clinical investigation (Rosales-Mendoza et al. 2015); however, more drugs and vaccines are needed to move from discovery into the development pipeline because of the high rate of drug attrition in clinical development and the potential for post-approval failures (Koul et al. 2011). In addition, it will also be challenging to ensure the proper access of the vaccines to patients in the resource poor countries where these are more needed (Kaufmann et al. 2010; Koul et al. 2011). Hence, their cost-effective production would be of prime importance.

Plant biotechnology has been employed in the development of cost-effective vaccines against TB. Several studies reporting the expression of different antigens against TB has been recently summarised by Rosales-Mendoza et al. (2015). Various antigens that have been expressed in plants include Ag85B, ESAT-6, Mtb72F, MPT83 and MPT64. Mostly these reports use either transient or stable nuclear transformation. An effective antigen-based TB vaccine should consist of multi-antigens along with adjuvant that can enhance cytotoxic T-lymphocyte (CTL) responses. Hence, it may be needed to diversify the current approaches to obtain more improved vaccine formulations, which may include expressing multiple open reading frames through polycistronic arrangements in the chloroplasts.

## Hepatitis

Hepatitis is the inflammation of liver characterized by symptoms such as jaundice, extreme fatigue, nausea and vomiting. Three main hepatitis types A, B and C are caused by infection with hepatitis A, B and C viruses, respectively. Globally, it is estimated that approximately 1.5 million people die each year from various forms of viral hepatitis (WHO 2015c), mostly by hepatitis B and C. Infection rate is very high among people in developing countries and 90 % infected individuals are children. Although, hepatitis A does not cause chronic liver disease, it can lead to high mortality due to acute liver failure. Hepatitis B is the most severe form of hepatitis and WHO estimates that worldwide 240 million people are chronically infected with hepatitis B virus and 780,000 people die every year due to acute or chronic illness (WHO 2015d). In case of hepatitis C, approximately 130–150 million people globally are chronically infected and half million people die each year from hepatitis C-related liver diseases (Lozano et al. 2012). Effective vaccines are available against hepatitis types A and B. For immunization against hepatitis type A, vaccine consists of inactivated or live attenuated formulations. In case of hepatitis B, antigen-based vaccine containing surface antigen of hepatitis virus (HBsAg) is available. Both these vaccines have been very effective in fighting against hepatitis A and B. There are no vaccines against other types of hepatitis. For hepatitis C, antiviral therapy with interferon and ribavirin was successful in curing approximately half of infected patients but sometimes caused severe side effects (Fried 2002).

Although, subunit vaccine against hepatitis B consisting of small surface antigen of hepatitis B virus (S-HBsAg) has proved highly effective and safe, there is still room for improvements regarding low-cost platforms and specific antigens (Rybicki 2014). Lack of response from certain populations to the current vaccine antigens also urges the development of third generation vaccines, consisting of middle (M-HBsAg) and/or large (L-HBsAg) surface antigens, which contain the strongly immunogenic preS1 and/or preS2 domains (Rybicki 2014). Plants can be used to produce an oral vaccine against hepatitis B that can circumvent use of needles for injectable delivery. HBsAg has been expressed in plants and its immunogenicity has been evaluated in animal models. For the first time Mason et al. (1992) expressed HBsAg in plants in the form of VLPs. Since then there have been many reports on plant-based production of antigens against hepatitis B using different approaches as summarised by Pniewski (2014). Few recent reports also show the expression of antigens against hepatitis B. In one of these reports, oral immunogenicity of the HBsAg synthesized in the tubers of marker-free potato plants was demonstrated (Rukavtsova et al. 2015). In this study, mice fed with transgenic potato tubers, showed an increase in the level of HBsAg antibodies significantly above the protective value, which was maintained for 1 year after the immunization. Peyret et al. (2015) opted for a novel strategy that helped in the display of whole proteins on the surface of hepatitis B core (HBc) particles. This strategy, named as tandem core, is based on the production of the HBcAg dimer as a single polypeptide chain by tandem fusion of two HBcAg open reading frames. In case of hepatitis C and A, few reports show some promising approaches. For instance, Nemchinov et al. (2000) expressed a synthetic hypervariable region 1 (HVR1)-derived peptide called R9, a potential neutralising epitope of HCV derived from the envelope protein E2, fused to the C-terminal of the B subunit of cholera toxin (CTB). The plant-derived antigen reacted with sera from HCV infected individuals and mice immunized with crude plant extracts produced anti-HVR1 antibodies. Subsequently, many research groups expressed R9, E1 and E2 proteins from HCV in plants (for review see Rybicki 2014). Recently, hepatitis C virus core protein in oil seeds of *Brassica napus* was expressed (Mohammadzadeh et al. 2015). Couple of reports also show the feasibility of antigen-based plant-produced vaccine candidates against hepatitis A (Chung et al. 2011, 2014).

## Pneumonia

Pneumonia is a form of acute respiratory infection that affects the lungs. Pneumonia is the leading cause of death in children worldwide, accounting for 15 % of all deaths of children under 5 years and killing an estimated 922,000 children in 2015 (WHO 2015e). It is estimated that 99 % of cases occur in developing countries (Greenwood et al. 2007; Mulholland 2007). Most common and severe type is streptococcal pneumonia which is caused by *Streptococcus pneumoniae*. Infections due to this pathogen leads to hospitalization of more than 100,000 cases annually and is associated with approximately 60,000 cases of invasive diseases such as meningitis (Centre for Disease Control 2008). There are two types of pneumococcal vaccines available for use (Jackson and Neuzil 2008); pneumococcal polysaccharide vaccine (PPSV) and pneumococcal conjugate vaccine (PCV). PPSV vaccine contains 23 pneumococcal strains and is recommended by the CDC for adults over 64 years old and PCV13 vaccine contains 13 pneumococcal strains and is recommended for children between 2 months and 18 years old. However, these vaccines have been linked to certain side effects such as fever, severe local reactions (swelling, redness, pain at site of injection), irritability, drowsiness, restless sleep, vomiting, diarrhea, rash, decreased appetite, convulsions, asthma, pneumonia and sudden infant death syndrome (National Vaccine Information Centre 2015). In addition, pneumococcus has more than 90 serotypes, which vary by region. Current vaccines are effective against the serotypes contained in the vaccines but do not protect against all pneumococcal serotypes. Furthermore, the production platforms of these vaccines are complicated and relatively expensive, which makes these vaccines less affordable in low-income countries. Hence, to ensure safety and affordability, new strategies and platforms needs to be investigated.

## Liver and cervical cancer

Some infectious agents have been found to be either directly or indirectly related to various types of cancers. The infectious agents, mainly viruses associated with cancers have been reviewed by Pagano et al. (2004). Among the cancers related to infectious agents, two are liver and cervical cancer associated with hepatitis virus and human papillomavirus (HPV), respectively. Liver cancer is the fifth most frequently diagnosed cancer worldwide and the second most frequent cause of death due to cancer in men (Torre et al. 2015). Almost 85 % cases of liver cancer occur in developing countries (Ferlay et al. 2010). The rate of liver cancer is highest in East and South-east Asia and in Middle and Western Africa. In 2012, an estimated 782,500 new liver cancer cases and 745,500 deaths occurred, worldwide (Torre et al. 2015). Among primary liver cancers, hepatocellular carcinoma (HCC) is the major type and accounts for approximately 75–85 % of cases (El-Serag 2001). Among other risk factors, chronic infection with hepatitis B or C virus is associated with a high risk of developing HCC (Donato et al. 2002; Hassan et al. 2002). The high HCC rates in parts of Asia and sub-Saharan Africa may be largely due to high prevalence of chronic hepatitis B virus (HBV) infection, with over 5 % of the population in these regions chronically infected with the virus (Averhoff 2014). HBV and hepatitis C virus (HCV) account for an estimated 32 % of infection-related cancer cases (mostly liver cancer) in less developed countries and 19 % in more developed countries (De Martel et al. 2012).

Cervical cancer is overall seventh most common cancer and fourth most in women, worldwide (Ferlay et al. 2015). Cervical cancer accounted for 9 % of new cancer cases and 8 % of total cancer related deaths in 2008 throughout the world (Jemal et al. 2011). In developing countries, it is the second most commonly diagnosed cancer and third leading cause of cancer related deaths among females. It is estimated that more than 90 % of cervical cancer cases and deaths occur in developing countries (Torre et al. 2015). Several types of human papillomavirus (HPV) have been found to be causatively associated with cervical cancer (Munoz et al. 2003). Among these, HPV types 16 and 18 have been found to be involved in approximately 70 % of cervical cancer cases (Smith et al. 2007). HPV types are etiologically linked to some other cancers as well (Jemal et al. 2013). Targeting these infectious agents could help to reduce these cancers. However, due to high prices of vaccines, it is likely that the cancer burden is not going to change considerably in developing countries. Existing prophylactic vaccines against HPV are expensive and hence may not be available for mass immunization programs. In this context, alternate platforms need to be established for the production of vaccines against HPV. Many research groups have expressed vaccine candidates against different types of HPVs in plants. Mostly these are focused on L1, the major capsid protein of HPV. Plant-derived vaccines against HPV have been recently reviewed by Rosales-Mendoza and Govea-Alonso (2015).

## Need of next generation subunit vaccines

For the treatment of existing infections mostly chemotherapy is used. However, the development of drug resistant microorganisms is gradually making the treatment more challenging and the use of multidrug is not affordable in low-income countries due to elevated costs. Also, the costs and time needed for the development of new drugs for treatment can make the control of diseases more difficult in future. This problem can be solved by using vaccines against these diseases. Currently available vaccine formulations mostly consist of live attenuated or killed microorganisms. Although these vaccines have shown success and some diseases such as polio have been eradicated from most parts of the world, many issues still remain associated with these vaccines. For instance, there is need to maintain cooling chain, which is difficult to achieve in remote areas of developing countries. In people with compromised immune system due to malnutrition or HIV infection, live vaccines can lead to severe infection (Kaufmann et al. 2010), especially in developing countries where primarily children are undernourished and HIV incidence is high. In addition, the price of a vaccine is one of the most important factors affecting the ultimate utilization of vaccines in developing countries. Costs related to vaccine production and administration makes these vaccines difficult to use for mass immunization of people in developing countries, where these are needed most. Hence, there is a need to develop more effective subunit prophylactic as well as therapeutic vaccines against different infectious diseases. In those cases where subunit vaccines already exist, such as HPV or HBV vaccines, these are expensive and most people in developing countries cannot afford these vaccines (Waheed et al. 2012). Hence, it is preferable to develop prophylactic and therapeutic next generation subunit vaccines that should be safe, effective and low-cost.

## Plants as cost-effective platforms for vaccine production

Different aspects of plants as production systems for pharmaceuticals have been previously reviewed (Fischer et al. 2004; Daniell 2006; Bock 2007; Cardi et al. 2010; Clarke and Daniell 2011; Maliga and Bock 2011; Penney et al. 2011; Yusibov et al. 2011). Keeping in view the costs of multidrug treatments, drug resistance of microorganisms, hurdles in the development of new drugs and the affordability/availability of vaccines for the treatment of many infectious diseases, we highlight some promising prospects of plant-based systems for the cost-effective production of subunit vaccines against these diseases.

The main advantage of vaccine production in plants is the cost-effectiveness of the system. Plants can be grown at the site where needed and scaled up according to the requirements, making this system attractive for developing countries. In contrast, fermenter-based production systems require costly equipment, infrastructure and growth media, which ultimately affects the costs of products. Even if the original concept of edible vaccines lies far in reality and downstream processing has to be carried out, plants-derived vaccines could still be cost-effective, with up to 31 % estimated cost reduction (Rybicki 2009) at production level. Additionally, plants offer various unique advantages. These include proper folding of antigenic proteins, enhanced expression and the immunogenicity of plant-expressed antigens in animal models (Chan and Daniell 2015; Waheed et al. 2015). For vaccine production, very high expression can be pre-requisite. In literature, various reports are available that can be adopted for optimal expression of foreign proteins in plants (Streatfield 2007; Fahad et al. 2015; Waheed et al. 2015). Both nuclear and chloroplast genomes can be engineered to express vaccine antigens. Advantages of chloroplasts include very high expression of transgenes up to more than 70 % of total soluble protein (Oey et al. 2009; Ruhlman et al. 2010). Chloroplast-based expression also addresses regulatory concerns related to transgene containment because plastids are not spread via pollen in most plant species. Expression of multigenes as single operon is an advantage of chloroplasts that can be exploited to express multiple genes together (Lössl and Waheed 2011). This advantage is also very beneficial to develop multivalent vaccines against different infectious agents, which can reduce the costs related to their separate production and administration. Moreover, costs related to adjuvant production can also be evaded (Waheed et al. 2012). However, plastids cannot be used to express those vaccine antigens that need glycosylation for their activity. For those proteins that need glycosylation for their immunogenicity, nuclear transformation or viral-based delivery can be used.

The expression of transgenes in plants can be either stable or transient. Traditionally, plants are genetically modified with foreign genes stably integrated in their genomes. These genes are inherited to next generations. This technology has been optimized for stable integration of transgenes using different methods such as *Agrobacterium*-mediated and gene gun-mediated delivery of foreign genes. Stable transformation has been used to transform nuclear as well as plastid genomes of various plant species (Waheed et al. 2015). Stable genetic transformation has many advantages such as very high expression and optimized protocols for transformation of various plant species. One major advantage is the development of a stable genetic resource in the form of transgenic seeds, which can serve as a constant resource to grow the transgenic plants and extract the proteins. However, costs related to stable transformation can be high and the process is also time consuming. Arguably, costs related to stable transformation are just one time that are invested during the initial development of transgenic lines and stable genetic resource. Later, the seeds can be grown at any site with minimal resources and labour. In contrast, in case of transient system, the plants need to be transformed every time which requires the use of resources such as media and cultures and labour each time. On the other hand, transient technology using either *Agrobacterium* and/or viral vectors is robust, quick and easy to manipulate (Komarova et al. 2010). In recent years, interest in transient expression is increasing due to containment of the system and rapid production that are attractive features for industrial scale production and approval of the expressed products. Hence, transient expression carried out in contained facilities fulfils the good manufacturing practices as well as quick expression can evade the time consuming stable transformation (Komarova et al. 2010; Fischer et al. 2012). Although, both transient and stable expression systems have their own advantages, for vaccine production stable transformation and establishment of stable genetic resource can be important for on-site production.

A major advantage of expression of vaccine antigens in plants is that the plants can be consumed as raw materials. Thus the vaccine antigens expressed in plants can be used for oral administration (Jin and Daniell 2015). This potential of plant-based vaccines is vital in cost reduction because the costs related to downstream processing and purification can be circumvented. Production of edible vaccines permits the use of non-processed plant materials that are resistant to digestive enzymes in the gastrointestinal tract due to bioencapsulation through cell wall. Also, edible vaccines administered via oral route can be used to treat autoimmune diseases and to induce oral tolerance to allergens (Sack et al. 2015). An additional advantage of expression of bioencapsulated pharmaceuticals is the stability of expressed antigens within the plant tissues. In this way, vaccine antigens can be stored at elevated temperatures for longer times and also transported without the need of cooling chain. Vaccine candidates can be expressed in seeds that may remain stable for several years at ambient temperatures and can be effectively delivered to gut-associated lymphoid tissues without degradation because of double packaging into cell wall and protein bodies (Takaiwa 2011; Sack et al. 2015). Thus this strategy may provide additional advantages in terms of storage and cold chain-free delivery of seed-based edible vaccines. However, it is important to assure the quality and quantity of pharmaceuticals produced in raw plant materials and to prove the antigenic properties of vaccine candidates in animal models.

In recent past, industrial interest has increased in plant-produced products (Paul et al. 2015). One of the plant-produced therapeutic products, the enzyme glucocerebrosidase has been synthesized and marketed by Protalix (http://www.protalix.com/glucocerebrosidase.html). Medicago has started a phase II clinical trials for a plant-derived VLP quadrivalent influenza vaccine (https://clinicaltrials.gov/ct2/show/NCT02236052?-term=plant-based+vaccine&rank=2). Plants are gradually becoming an attractive platform for the production of pharmaceuticals and many companies are taking interest in these systems because of their low-costs. Plant-based systems can be more attractive to newly established companies or those located in resource poor countries that can utilize the cost-effective production to generate good amount of revenue. This can help to boost the economy of developing countries. Government bodies can also play a significant role to strengthen local pharmaceutical companies to establish plant-based platforms for highly needed vaccines. Production of vaccines locally will exclude the costs related to transportations and cooling chain maintenance. Consequently, vaccines may become available at low costs in these countries.

## Conclusions

Infectious diseases have more burden in developing countries and current preventive and treatment strategies have not been successful to effectively reduce this burden. Hence alternatives such as development of effective vaccines are needed to strengthen the control strategies. In addition, efforts are needed to reduce the costs of existing vaccines to make these affordable for people in developing countries. For this purpose plants can be used as a cost-effective platform. Various antigens against different diseases have been expressed in plants. Increasing industrial interest in plant-made pharmaceuticals is also promising which can promote the availability of the pharmaceuticals to low and middle income countries where disease burden is high.
